# Mini Review: Socio-Cultural Influences on the Link Between ADHD and SUD

**DOI:** 10.3389/fpubh.2019.00173

**Published:** 2019-06-26

**Authors:** Ortal Slobodin, Cleo L. Crunelle

**Affiliations:** ^1^Department of Education, Ben-Gurion University, Be'er Sheva, Israel; ^2^Department of Psychiatry, Vrije Universiteit Brussel, Universitair Ziekenhuis Brussel, Brussels, Belgium; ^3^Toxicological Center, Antwerp University, Antwerp, Belgium

**Keywords:** ADHD, culture, ethnicity, socio-economic status, SUD

## Abstract

Attention-deficit/hyperactivity disorder (ADHD) is a risk factor for the development and persistence of substance use disorders (SUD). The prevalence of ADHD in patients with SUD varies across countries and cultures. So far, cross-cultural variance in ADHD prevalence rates among SUD patients was mainly ascribed to methodological differences between studies, leaving the role of societal and cultural practices in the ADHD-SUD link hardly acknowledged. The aim of the present mini review is to address this gap in the literature by providing evidence for the effect of socio-cultural practices on the ADHD-SUD link and suggesting directions for future research. To achieve this goal, we map the influence of socio-cultural factors on the ADHD-SUD link along three lines of research. The first line is concerned with the role of socio-cultural factors in the recognition, diagnosis and treatment of childhood ADHD. The second line of research is concerned with socio-cultural influences on substance use (e.g., religion, ethnic identity, acculturation, and socio-economic status). Finally, we describe potential socio-cultural factors which may operate as mechanisms for reducing or increasing access to substance abuse treatment (e.g., ethnic disparities in health care utilization). Identifying socio-cultural influences on the ADHD-SUD link may provide further insight into the bidirectional association between ADHD and SUD in different contexts and encourage future research in the field.

## Introduction

Attention-deficit/hyperactivity disorder (ADHD) and substance use disorders (SUDs) are inextricably intertwined. It has been estimated that one quarter of those suffering from SUD have comorbid ADHD (1). Substance using individuals with ADHD have earlier onset of substance abuse, higher self-rated impairment across several domains of daily life, and worse prognosis in treatment compared to non-ADHD substance abusers (2, 3). To date, the mechanisms underlying the bidirectional association between ADHD and SUD are not entirely clear (4). Since both ADHD and SUD are known to be familial disorders [heritability estimate of ADHD is approximately 80% (5) and that of SUD is 40–80%, depending on substance (6, 7)], genetic contributions (8) as well as exposure to parental SUD (9) were suggested as possible explanations for their co-morbidity. Based on familial risk analysis of two longitudinal studies of probands aged 6 to 17 years, Yule et al. (10) suggested various pathways through which SUD may be transmitted in ADHD families. These include the risk associated with SUD itself, the risk conferred by ADHD, and the risk conferred by the co-segregation of ADHD and SUD. In a recent review of family, twin, and molecular ADHD genetic studies, Faraone and Larsson (11) argued that the facts that twin estimates of ADHD's heritability are <100% and that this heritability is explained by single nucleotide polymorphisms in regulatory regions rather than coding regions (12), assert quite strongly that environmental factors and gene by environment interactions account for much of ADHD's etiology.

Another explanation for the ADHD-SUD link is that ADHD-related symptoms, in particular impulsivity, lead to trying substances (13, 14). In addition, SUD and ADHD may share similar structural and functional deficits (15), particularly in relation to dopamine transmission (16, 17).

In addition to behavioral, neuropsychological, and genetic pathways, the association between ADHD and SUD is greatly influenced by socio-cultural factors. Given that drug use is often much more than a simple action performed in order to experience a physical or psychological reaction (18), culturally-relevant factors including social activities, availability, potential legal consequences, economic disparity, inadequate implementation of policies related to drug use and high-risk groups may all have a great significance to the understanding of the link between ADHD and SUD in a certain ethnic population (19).

International research revealed a wide variance in the prevalence of ADHD in SUD patients across countries and cultures (20, 21), even when controlling for diagnostic procedure (22). Among treatment seeking SUD patients, ADHD prevalence ranged between from 2% in substance abusing Icelandic adolescents (23) to 83% in Japanese methamphetamine and inhalant abusers (24). So far, cross-cultural variance in ADHD prevalence rates among SUD patients was mainly ascribed to differences in the types of abused substances and their availability, the setting (e.g., outpatient vs. inpatient), different sample sizes, and differences in the diagnostic procedure (22). While most of these studies focused on characteristics intrinsic to ADHD (e.g., impulsivity, novelty seeking, disinhibition) as risk factors for SUD, the role of societal and cultural practices in the ADHD-SUD link was hardly addressed. The aim of the present review was, therefore, to address this literature gap by describing socio-cultural influences on the ADHD-SUD link.

The associations between ADHD and SUD are usually examined in treatment-seeking SUD patients (1). Therefore, identifying socio-cultural aspects of the link between the two disorders should be investigated across the developmental trajectory that begins with ADHD and progresses to SUD (25). To achieve this goal, we map the influence of socio-cultural factors on the ADHD-SUD link along three lines of research: (1) The role of socio-cultural factors in the recognition, diagnosis and treatment of ADHD. (2) Socio-cultural influences on substance use (e.g., religion, acculturation, ethnic identity, socio-economic status), and (3) Socio-cultural factors that may operate as mechanisms for reducing or increasing access to substance abuse treatment (e.g., ethnic disparities in health care utilization).

## Socio-cultural Factors in ADHD Care

Untreated childhood ADHD is a risk factor for the development of SUD (26). Therefore, cultural and ethnic diversity in the recognition, diagnosis and treatment of childhood ADHD may explain some of the obtained cross-cultural differences in the prevalence of the syndrome among SUD patients.

Culturally relevant factors, such as norms, medical approaches, beliefs, and values influence the way members of various cultural groups view and respond to problematic behavior in children (27). Cross-cultural studies, mostly from the U.S (28, 29) but also from Europe (30, 31) and Israel (32, 33), have shown that ethnic minority children are less likely to be recognized and treated for ADHD than their non-minority counterparts.

A range of perceptions and attitudes may be responsible for avoiding or delaying help seeking for ADHD, including limited knowledge about ADHD (34), fear of stigmatization (35), mistrust in school or health care systems (36), and having a higher threshold for behavioral tolerance before seeking assessment (37). In some cultures, for example, the Muslim and Christian Lebanese, hyperactivity and/or impulsivity in boys can be endorsed as typical by parents and viewed as gender preferred behavior (38). In addition, organizational, economic and environmental factors, such as language difficulties, limited insurance coverage, limited access to health care services and lack of culturally competent providers serve as barriers to adequate diagnosis and treatment among ethnic minorities and low socio-economic populations (39).

Socio-cultural barriers to accurate diagnosis and treatment of childhood ADHD may be further exacerbated in adult ADHD, especially among individuals in the criminal justice system (40). To qualify for ADHD as an adult, one must have had it as a child, making the identification of ADHD in adults a complicated task for both patients and professionals (41). Moreover, the presence of co-morbid psychiatric disorders among youth and adults with ADHD often confounds and influences treatment options (42). Currently, very little is known about cultural or ethnic diversity in adult ADHD (43). According to the American National Institute of Mental Health (44), adults of an ethnic minority background are less likely to be diagnosed with ADHD than non-minority groups. However, the efficacy of ADHD treatment in reducing psychiatric morbidity, transport accidents and criminality emphasizes the academic and clinical imperative of studying ethnic diversity in ADHD care among various ethnic and cultural groups (45, 46).

Despite growing concerns about the under-diagnosis and under-treatment of mental health problems among certain socio-cultural groups (47), cultural, racial, and language biases may also lead to over-identification of ethnic minority groups as disabled and so disproportionately over-represented in special education (48, 49).

While cultural diversity may be partly attributed to better screening, knowledge and awareness in Western cultural contexts, these factors may interact with environmental risk factors to affect ADHD risk (50). For example, immigration history and low socio-economic status have been associated with higher exposure to environmental risk factors (e.g., prenatal substance exposures, nutritional factors, and psychosocial stressors), that may increase the risk for ADHD (51). Taken together, the above-mentioned findings suggest that socio-cultural factors pertain every phase of ADHD care, affecting the likelihood that ADHD will be recognized, diagnosed and treated. Such differences across cultures and contexts, which may also be associated with geographical differences in environmental risk factors, may have a large effect on the risk of develop SUD in adulthood.

## Socio-cultural Predictors of Drug Use and Abuse

Cultural norms, beliefs and attitudes, including ideologies, practices, and symbols, have a central role in forming the expectations of individuals about potential problems they may face with drug use. Such cultural norms are represented in legends, proverbs, anecdotes, and jokes, as well as in fiction and entertainment (e.g., “alcohol warms the soul,” “we do not drink, but only cure ourselves”) (52).

Among the multiple influences of socio-cultural aspects on substance use and abuse, this section focusses on the role of three cultural processes which were particularly associated with delinquency and substance use; religious affiliation, acculturation and ethnic identity.

Religious affiliations often play a protective role against drug use (53, 54). Religious involvement may inhibit adolescent risk behavior by altering behavior-influencing values or by functioning as an external control factor (55). Affiliation with religions that forbid alcohol consumption was also associated with less use of alcohol (56). Studies of European school and college students reported that first and second-generation immigrants from Muslim majority countries were less likely to drink (57, 58) and that their low levels of drinking influenced drinking overall at the schools (59).

Although lower prevalence of SUD among traditional and religious cultures may indicate compliance with socio-cultural rules or greater social control, it may also indicate a society's failure to acknowledge problems of addictions (60). For example, denial of SUD has been widely noted in Ultra-Orthodox Jews, who tend to hold the myth that Jews cannot have addictions (61). This belief results in leaders of the community failing to address the problem and discourages health professionals to conclude the diagnosis of SUD in this culture (62, 63).

Acculturation, the degree to which an individual identifies with his or her native culture, may also be related to substance use and abuse. The association between acculturation and substance use is equivocal, but overall greater levels of acculturation are associated with increased levels of substance use (64, 65). Acculturation was identified as a risk factor for alcohol use among immigrants and their children from countries with Muslim majorities in European studies (66, 67). Acculturation may increase the risk for substance use in several possible ways. According to the stress hypothesis (68), acculturated youth are particularly vulnerable to negative psychological, social, and academic outcomes due to increased psychosocial stressors, which may contribute directly and indirectly to substance use. These stressors include psychological problems such as anxiety, depression, somatic complaints, social problems, attention problems, delinquency, and aggressive behavior as well as social problems like marginalization, discrimination, oppression, and family conflicts (69, 70). Acculturation may also be associated with greater availability and accesses to substances (71) and with a greater susceptibility to peer pressure (72). In addition, acculturation is often compounded by a concomitant decrease in religiosity (73), thereby liberating individuals from cultural norms that prohibit alcohol or drug use (74, 75). Finally, acculturation can be associated with SUD because it involves a breaking of traditional, communal, spiritual, and physical circles that previously protected from delinquency. For example, several authors linked the disappearance of American Indian and Alaska Native traditions to increased substance and alcohol abuse (76, 77).

In addition to acculturation, ethnic identity may also be associated with substance use in certain cultural groups. However, its role in predicting substance use remains elusive (78). Strong ethnic identity, pride and affirmation could help immigrant adolescents to overcome hardships of immigration (79), thus contributing to better adaptation (80). On the other hand, strong ethnic identification could be a result of perceived discrimination (81), as the immigrant feeling rejected from the new host society seeks a stronger connection with his or her heritage culture. Therefore, ethnic identity would be associated with adverse outcomes. To date, studies about the relationship between ethnic identity and substance abuse revealed mixed results (82, 83).

Socio-economic status (SES) has various contradictory effects on the ADHD-SUD link. Lower SES is associated with both an increased ADHD and SUD incidence [(84), for meta-analysis]. Lower SES is also associated with reduced access to resources (e.g., proper food, housing, education), unhealthy life style, heightened family conflicts (85, 86), and higher exposure to toxic chemicals and pollution (87), which were all previously linked to increased risk for ADHD (88, 89). Low-income communities often have fewer services available, lack of health insurance coverage, limited access to mental health care and potentially increasing mental health concerns for residents in those communities (90, 91). Nevertheless, increased socio-economic status may also relate to increased access and availability of various substances (92, 93) including prescription drugs (94, 95). In regard to stimulant use, socio-cultural factors may have various effects (96). On the one hand, ethnic minorities and low-SES populations are less likely to be prescribed with stimulants and to adhere with treatment (97), therefore, more likely to develop SUD compared to those treated for the disorder. On the other hand, high SES is related to higher risk for the misuse of stimulants (giving away, trading, or selling of prescription medication (94, 95, 98).

## Socio-cultural Aspects of SUD Treatment

The link between ADHD and SUD is usually investigated in treatment-seeking SUD patients (1). Therefore, socio-cultural influences on SUD treatment may have an important contribution to cultural diversity in the observed associations between the two disorders.

Overall, studies suggest that racial and ethnic minority groups are less likely than non-minority groups to enter, receive, and complete treatment for SUD (99). Ethnic disparities were identified in health care policies and regulations, provider level factors, the operation of the community system, and patient level factors (100). While the causes of the observed service gap are complex, there is an increasing recognition that poor cultural competence and/or insufficient language services are one the main reasons for ethnic minorities' underutilization of SUD treatment services (101, 102).

Treatment preference is a relatively neglected area of study in service disparities (103). Preliminary findings suggest that ethnic minorities prefer individual sessions for the treatment of SUD, probably due to the fear of negative stigma (104, 105).

Interestingly, differential referral from schools, healthcare, and government organizations may operate as mechanisms moderating access to SUD treatment (106). For example, a study of the National Survey on Drug Use and Health (*N* = 25,159) suggested that treatment programs that are mandated by the criminal justice system might provide access to individuals resistant to care (107).

Little is known about the role of socio-cultural factors in predicting SUD treatment adherence and outcomes. Currently, cross-cultural studies comparing treatment efficacy of intervention to reduce substance use between ethnic minorities and non-minority groups are scarce. A meta-analysis that compared the impact of cognitive behavioral therapy (CBT) in reducing substance use between studies with a predominantly White sample and studies with a predominantly Black and/or Hispanic sample (*N* = 17 studies), indicated that CBT, when compared to a comparison group, was equally effective in minority and non-minority studies. However, when comparing pre-posttest effect sizes from groups receiving CBT between minority and non-minority studies, CBT's impact was significantly stronger in non-minority studies (108). Several studies examining culturally competent interventions for SUD have highlighted the importance of cultural adaptation [(109, 110), for meta-analyses] and flexible, client-centered approach (111) in improving treatment outcomes. A meta-analysis by Smith et al. (112) revealed that culturally adapted interventions were frequently more effective than standard interventions and that most effective interventions included more adaptations in terms of language, content, goals, methods, context of services, etc. (113). Likewise, a recent meta- analysis (*N* = 7 studies) including CBT, educational, individual and family interventions for SUD, concluded that culturally sensitive treatments were associated with significantly larger reductions in post-treatment substance use levels relative to their comparison conditions (110). Nevertheless, further research is needed to determine the efficacy of culturally responsive practices in reducing ethnic disparities in SUD treatment (114).

## Conclusions

This review highlights the role of socio-cultural aspects in the link between ADHD and SUD. These aspects include cultural factors such as perceptions of normal and abnormal behavior, behavioral norms, and attitudes and knowledge about mental health problems (37, 115) as well as factors related to the utilization of mental health services among certain populations (102). Interpretation of the studies integrated in this review is hindered by the limited number of included studies, small sample sizes, and ethnic heterogeneity and variability in comparison conditions across studies (110). Therefore, further large-scale research in needed in order to determine the efficacy of culturally responsive practices in reducing ethnic disparities in SUD treatment (114).

Continued research on socio-cultural aspects along with biological aspects is required to understand the interplay of all factors in the ADHD-SUD link. Understanding the role of culture and context in the ADHD-SUD link may not only shed light on the current enormous variance in the prevalence of ADHD in SUD patients across cultures (21), but also assist in early and accurate detection of ADHD in SUD patients.

## Author Contributions

OS and CC contributed equally to the literature search, literature analysis, data integration, and writing the review manuscript.

### Conflict of Interest Statement

The authors declare that the research was conducted in the absence of any commercial or financial relationships that could be construed as a potential conflict of interest. The reviewer CB declared a past co-authorship with the authors to the handling editor.
